# Hand Hygiene Practices and Promotion in Public Hospitals in Western Sierra Leone: Changes Following Operational Research in 2021

**DOI:** 10.3390/tropicalmed8110486

**Published:** 2023-10-27

**Authors:** Matilda N. Kamara, Sulaiman Lakoh, Christiana Kallon, Joseph Sam Kanu, Rugiatu Z. Kamara, Ibrahim Franklyn Kamara, Matilda Mattu Moiwo, Satta S. T. K. Kpagoi, Olukemi Adekanmbi, Marcel Manzi, Bobson Derrick Fofanah, Hemant Deepak Shewade

**Affiliations:** 1 College of Medicine and Allied Health Sciences, University of Sierra Leone, Freetown 00232, Sierra Leone; lakoh2009@gmail.com (S.L.); samjokanu@yahoo.com (J.S.K.); 2 Ministry of Health and Sanitation, Government of Sierra Leone, Freetown 00232, Sierra Leone; christy.conteh@yahoo.com (C.K.); sattasylvia@yahoo.com (S.S.T.K.K.); 3 US Center for Disease Control and Prevention, Freetown 00232, Sierra Leone; rugiatuzkamara@gmail.com; 4 World Health Organization Country Office in Sierra Leone, Freetown 00232, Sierra Leone; ibrahimfkamara@outlook.com (I.F.K.); derrickfbob@gmail.com (B.D.F.); 5 Republic of Sierra Leone Armed Forces, Government of Sierra Leone, Freetown 00232, Sierra Leone; mmmoiwo@gmail.com; 6 Department of Medicine, University of Ibadan, Ibadan 200005, Nigeria; kemiosinusi@gmail.com; 7 Department of Medicine, University College Hospital, Ibadan 200005, Nigeria; 8 Independent Researcher, 5000 Namur, Belgium; m.manzi449@gmail.com; 9 Division of Health Systems Research, ICMR-National Institute of Epidemiology (ICMR-NIE), Chennai 600077, India; hemantjipmer@gmail.com

**Keywords:** healthcare-associated infections (HAIs), hand hygiene self-assessment framework (HHSAF), infection prevention and control (IPC), Structured Operational Research Training Initiative (SORT IT), hand hygiene training, IPC in hospital

## Abstract

Hand hygiene is the most important intervention for preventing healthcare-associated infections and can reduce preventable morbidity and mortality. We described the changes in hand hygiene practices and promotion in 13 public hospitals (six secondary and seven tertiary) in the Western Area of Sierra Leone following the implementation of recommendations from an operational research study. This was a “before and after” observational study involving two routine cross-sectional assessments using the WHO hand hygiene self-assessment framework (HHSAF) tool. The overall mean HHSAF score changed from 273 in May 2021 to 278 in April 2023; it decreased from 278 to 250 for secondary hospitals but increased from 263 to 303 for tertiary hospitals. The overall mean HHSAF score and that of the tertiary hospitals remained at the “intermediate” level, while secondary hospitals declined from “intermediate” to “basic” level. The mean score increased for the “system change” and “institutional safety climate” domains, decreased for “training and education” and “reminders in the workplace” domains, and remained the same for the “evaluation and feedback” domain. Limited resources for hand hygiene promotion, lack of budgetary support, and formalized patient engagement programs are the persistent gaps that should be addressed to improve hand hygiene practices and promotion.

## 1. Introduction

Healthcare-associated infections (HAIs), defined as infections that occur during patient care, result in avoidable deaths or prolonged hospitalization for millions of people worldwide [1,2,3]. HAIs increase healthcare costs and contribute to the development of antibiotic-resistant infections [1,4]. Hence, preventing HAI is an important strategic intervention for patients’ safety and the quality of health care [5]. Hand hygiene is the single most important strategy for reducing the spread of HAI and antimicrobial-resistant pathogens [6,7,8].

The World Health Organization (WHO) has identified hand hygiene as the core indicator of patients’ safety. In 2010, WHO recommended the hand hygiene self-assessment framework (HHSAF) tool to assess the progress of healthcare facilities in hand hygiene promotion and practices [9]. Many hospitals in low- and middle-income countries (LMICs) have implemented the HHSAF tool, identified gaps, and developed actions for improvements [8].

Sierra Leone, a low-income country on the West Coast of Africa, established its first national policy on infection prevention and control (IPC) practices following the adversity of the largest Ebola outbreak to date during 2014–2016 [10,11]. Sierra Leone has been collecting data annually to assess hospitals’ compliance with hand hygiene practices and promotion using the WHO HHSAF tool.

In 2021, the Special Programme for Research and Training in Tropical Diseases (TDR) based at WHO (Geneva) led a Sierra Leone national Structured Operational Research and Training Initiative (SORT IT) course focusing on antimicrobial resistance [12]. Through this course, we conducted an operational research study in 13 public hospitals in the Western Area of Sierra Leone using the HHSAF tool [13]. We observed an “intermediate” level of hand hygiene promotion and practice (HHSAF score 251–375) and challenges such as the lack of dedicated budgets to support hand hygiene activities and a formalized patient engagement strategy [13]. Following this operational research, a hand hygiene improvement initiative was launched in 2022. This included engagement of stakeholders at the national and hospital levels, actions to formalize patient engagement, and systematic audits of hand hygiene promotion resources.

In this first-of-its-kind study from Sierra Leone, we aimed to describe the changes in hand hygiene practice and promotion in 13 public hospitals in the Western Area of Sierra Leone following the implementation of recommendations from the previous operational research [13]. Specifically, among the 13 public hospitals in the Western Area of Sierra Leone, before (May 2021) and after (April 2023) the implementation of the recommendations from an operational research project, we described the changes in (i) compliance with hand hygiene practice and promotion, overall and stratified by hospital type and domains (systems change, training and education, evaluation and feedback, reminders in the workplace, and institutional safety climate) and (ii) performance against each indicator under the various domains of the HHSAF tool.

## 2. Materials and Methods

### 2.1. Study Design

This was a “before and after” observational study using two routine cross-sectional assessments of hand hygiene practices and promotion conducted by IPC teams.

### 2.2. Study Setting

#### 2.2.1. General Setting

According to the 2015 population census, an estimated 8 million people live in Sierra Leone, of which 42% are under 15 years of age [14]. It is a country with an income per capita of $470 in 2018 [15,16,17]. There are five geographic regions in the country, including the Western Area (urban and rural), which is the most densely populated region with about 1.5 million people and includes the capital, Freetown [14].

#### 2.2.2. Specific Setting

The public health system in Sierra Leone is divided into primary, secondary, and tertiary care levels. The peripheral health units provide primary care, district hospitals provide secondary care, and regional/national hospitals provide tertiary care. There are 25 secondary and tertiary level hospitals in Sierra Leone, 13 of which (including six secondary and seven tertiary hospitals) are in the Western Area [17].

The technical leadership for IPC at each hospital is provided by nurses designated as an “IPC focal person”. Every ward or unit in a hospital has an “IPC link nurse” who is responsible for overseeing the IPC practice in the ward/unit. The data for the HHSAF are collected annually from the “IPC focal person”.

### 2.3. Study Population

The study population includes all 13 public hospitals in the Western Area (urban and rural districts) of Sierra Leone.

### 2.4. HHSAF Tool

The HHSAF has been developed and validated by WHO as a method of assessing different aspects of how a hospital is addressing hand hygiene at an institutional level [9].

There are five domains in this tool: (i) system change, (ii) training and education, (iii) evaluation and feedback, (iv) reminders in the workplace, and (v) institutional safety climate [9]. The “system change” domain assesses hand hygiene resources (such as alcohol-based hand rub, soap, water, and single-use towels), the sink-to-bed ratio, and systems for implementing hand hygiene practices. The “training and education” domain assesses the skills of healthcare workers in conducting hand hygiene training and the processes and systems that support this training. The “evaluation and feedback” domain assesses audits, monitoring of hand hygiene compliance, and systematic feedback on hand hygiene. The domain “reminders in the workplace” assesses the availability of hand hygiene promotional materials such as posters, campaign screensavers, badges, and leaflets. The “institutional safety climate” domain has indicators of institutional culture of teamwork, leadership support, patient engagement, and designation of hand hygiene role models or champions.

Once a year, all public hospitals are expected to generate a total score using the HHSAF tool, identify gaps, and take necessary actions. Each domain has various components that are scored [9]. Depending on the total scores in each domain, hospitals are categorized into four hand hygiene levels: “inadequate” (0–125), “basic” (126–250), “intermediate or consolidation” (251–375), or “advanced or embedding” (376–500) (Table S1).

The IPC focal person coordinates this and reports to the national IPC coordinating unit. Any hospital with an advanced level additionally provides answers to questions under the “leadership” domain. Within each domain, based on the score, we classified the performance as excellent (>70), good (51–70), poor (35–50), or very poor (<35) [13].

### 2.5. Information from Previous Operational Research, Dissemination Activities, Decisions, and Actions

During the final SORT IT module (in May 2021) on “communicating research findings” for the previous operational research [13], we mapped the potential stakeholders for advocacy and dissemination. We prepared the following advocacy materials: a handout, detailed technical presentation (PowerPoint and video), lightening presentation (PowerPoint), and elevator pitch [18]. We used these resources for the dissemination of findings and advocacy to stakeholders for improvement in hand hygiene promotion and practice.

**Figure 1:**
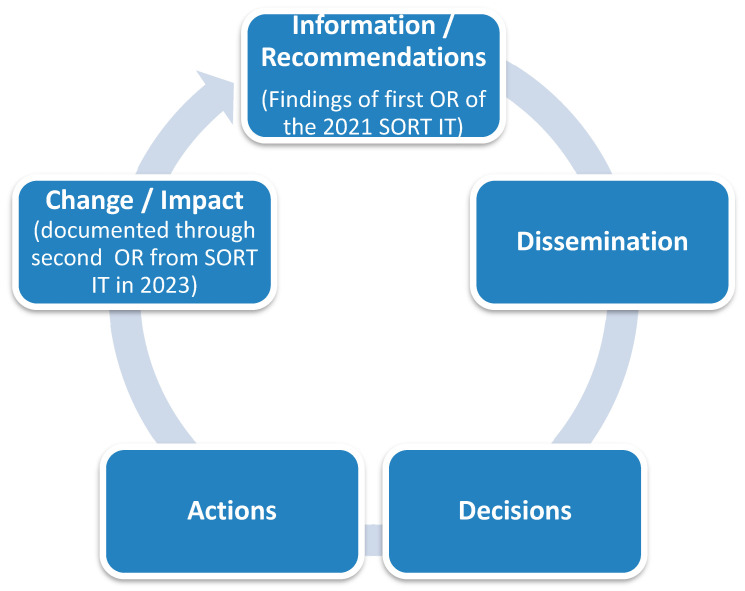
“Information-Dissemination-Decisions-Action-Change” cycle. OR—operational research; SORT IT—Structured Operational Research and Training Initiative.

Keeping the framework depicted in Figure 1, we describe the information (and corresponding recommendations) from the previous operational research in May 2021 [13] in Table 1. The dissemination activities, decisions taken, and details of how many of them have been acted upon as of January 2023 are in Table 2 and Table 3.

### 2.6. Data Variables and Sources of Data

Data from May 2021 (before) were already captured in our previous study [13]. In April 2023, we applied the same method for data collection in line with the previous operational research [13]. The data source was the routine cross-sectional assessment of hand hygiene promotion and practices by IPC teams using the HHSAF tool. The investigators facilitated this process during both the time periods, May 2021 (before) and April 2023 (after), during which they also acted as independent external observers.

General variables extracted/collected include type of hospital (secondary/tertiary), bed capacity (the total number of beds in a hospital), staff capacity (the total number of staff in a hospital), and the number of wards/units in a hospital. We collected the specific variables using the WHO HHSAF tool. These include hospital-wise total scores and domain-wise scores. We inferred the level of hand hygiene practices and promotion using the available cut-offs (Table S1).

### 2.7. Data Analysis and Statistics

Using the HHSAF tools, data for all 13 public hospitals from the previous operational research project (May 2021) were available in Microsoft Excel (Microsoft, Redmond, WA, USA). We single-entered data for April 2023 in EpiData Entry Client (version 4.6.0.6 EpiData Association, Odense, Denmark). We used mean (standard deviation) and frequency (proportions) to summarize the data from both time periods using EpiData analysis software (version 2.2.3.187, EpiData Association, Odense, Denmark).

### 2.8. Institutional Review Board Statement

Ethics approval for this study was granted by both the Sierra Leone Ethics and Scientific Review Committee of the Ministry of Health and Sanitation, Government of Sierra Leone (SLESRC No. 006/03/2023) and The International Union against Tuberculosis and Lung Disease Ethics Advisory Group, Paris, France (EAG number 04/2023).

## 3. Results

### 3.1. Hospital Characteristics

Comparing the hospital characteristics between May 2021 and April 2023, the same number of hospitals (*n* = 13) were assessed. There was an increase in the mean bed capacity from 111 in May 2021 to 132 in April 2023. The mean staff capacity decreased from 276 to 241 (Table 4).

### 3.2. Changes in the Mean HHSAF Score, Overall, by Hospital Type and by Domain Wise

**Figure 2:**
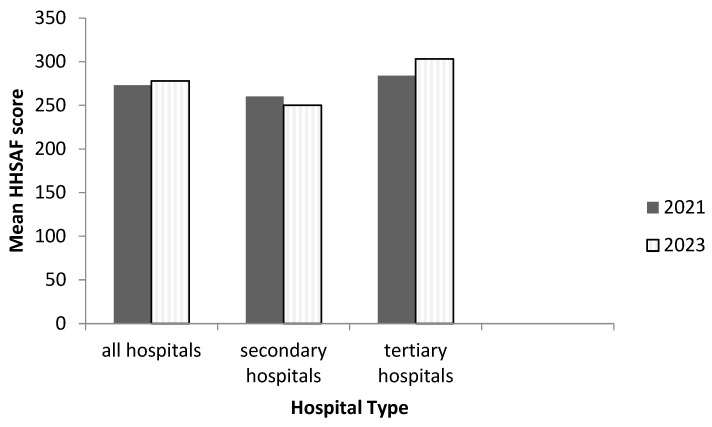
The mean HHSAF score of 13 public hospitals in the Western Area of Sierra Leone before (May 2021) and after (April 2023) the implementation of the recommendations from operational research [13].

The overall mean (±standard deviation) HHSAF score was 273 (±46) in May 2021 and 278 (±49) in April 2023. When compared to May 2021, in April 2023, it decreased from 278 (±67) to 250 (±46) for secondary hospitals but increased from 263 (±58) to 303 (±38) for tertiary hospitals (Figure 2). The overall mean HHSAF score and that for tertiary hospitals remained in the “intermediate or consolidation” level. The mean HHSAF score for secondary hospitals declined from “intermediate or consolidation” in May 2021 to “basic” level in April 2023.

We observed an increase in the mean score for the “system change” and “institutional safety climate” domains and a decrease for the “training and education” and “reminders in the workplace” domains. The score for the “evaluation and feedback” domain did not change (Table 5).

### 3.3. Change in Performance of Each Indicator under the Various Domains

We depict the distribution of total and domain-wise scores across the 13 public hospitals before (May 2021) and after (April 2023) in Table 6. For secondary hospitals (*n* = 6), there were 30 (6 × 5) “hospital-domain” instances of performance assessment, of which “very poor (red)” performance was observed in four instances in May 2021 when compared to six instances in April 2023. For tertiary hospitals (*n* = 7), there were 35 (7 × 5) hospital domain instances of performance assessment, of which “very poor (red)” performance decreased from 12 instances in May 2021 to zero instances in April 2023 (Table 6). If we observe domain-wise, at least one hospital reported a “very poor (red)” performance in May 2021 (Table 6). However, in April 2023, for the domains “system change” and “evaluation and feedback”, not a single hospital had a “very poor (red)” performance (Table 6).

#### 3.3.1. System Change

More hospitals in April 2023 (12, 92%) than in May 2021 (4, 46%) reported a continuous supply of alcohol-based hand rubs. The number of hospitals with soap at each sink increased from 10 (77%) in May 2021 to 13 (100%) in April 2023. While no hospital received a dedicated budget for the procurement of hand hygiene products in May 2021, a dedicated budget was provided to three in April 2023. We did not observe any major change in other indicators in this domain (Table S2).

As shown in Table 6, the documentation of excellent performance under this domain increased from three hospitals (two secondary hospitals and one tertiary hospital) to nine hospitals (four secondary hospitals and five tertiary hospitals). 

#### 3.3.2. Training and Education

Beyond the mandatory training for all professional categories at the commencement of employment, ongoing regular training was happening in six (46%) hospitals in May 2021, which reduced to four (31%) in April 2023. Ten (77%) hospitals in May 2021, compared to seven (54%) in April 2023, had a system in place to show that training has been conducted in the facilities. We did not observe any major change in other indicators in this domain (Table S2).

#### 3.3.3. Evaluation and Feedback

Regular ward audits for hand hygiene products were conducted at only two (15%) hospitals in May 2021 but none in April 2023. Knowledge of healthcare workers on hand hygiene indications was assessed in 11 (85%) hospitals in May 2021, and this decreased to 8 (62%) in 2023. None of the hospitals achieved more than 70% hand hygiene compliance in both time periods. No major change in other indicators was observed (Table S2).

#### 3.3.4. Reminders in the Workplace

The availability of all types of posters promoting hand hygiene across hospitals decreased in April 2023: this was the case for posters promoting hand hygiene indications (nine (69%) to four (31%)), posters depicting correct hand washing techniques (eight (62%) to six (46%)), and posters depicting the use of alcohol-based hand rub (eight (62%) to two (15%)). No major change in other indicators was observed (Table S2).

Four (31%) tertiary hospitals were rated “very poor (red)” in this domain in May 2021, but none in April 2023. On the other hand, the number of secondary hospitals rated as “very poor (red)” increased from none to four (31%) (Table 6).

#### 3.3.5. Institutional Safety Climate

Systems for the designation of hand hygiene champions were reported in seven (54%) hospitals in May 2021 compared with three (23%) hospitals in April 2023. Similarly, systems for the recognition and utilization of hand hygiene role models were available in five (39%) hospitals in May 2021 compared to one (8%) in April 2023 (Table S2). No major change in other indicators was observed (Table S2).

Five hospitals in May 2021 performed “very poorly (red)” in this domain compared to one hospital in April 2023 (Table 6).

## 4. Discussion

In this assessment of changes in hand hygiene practices and promotion in the Western Area of Sierra Leone following the implementation of recommendations from an operational research study, we found that the overall HHSAF score and level did not change across hospitals. While an improvement was observed in the mean score of tertiary hospitals, the level remained the same (“intermediate or consolidation”). In secondary hospitals, hand hygiene practices and promotion deteriorated from an “intermediate or consolidation” to a “basic” level. Furthermore, we observed an improvement in the performance of the “system change” and “institutional safety climate” domains but a deterioration in the performance of the “training and education” and “reminders in the workplace” domains.

Our study has strengths and limitations. To our knowledge, this study provides the first evidence of changes in hand hygiene practices before (May 2021) and after (April 2023) the implementation of the recommendations of an operational research study, and its findings have important policy implications for improving hand hygiene practice and promotion in Sierra Leone and the wider world. 

One of the limitations is that the HHSAF tool used in this study was not adapted to our local context, and, in the absence of adequate training, its indicators may be interpreted differently in routine practice. However, we overcame this through training and closely reviewing on a daily basis the data collected with the IPC focal persons. Going forward, we recommend the development of an annex document indicating the correct interpretation of this tool.

Second, we inferred changes based on public health significance, particularly if the HHSAF level changes. However, due to the small sample size (*n* = 13 hospitals), it was not appropriate to use statistical tests to assess the statistical significance of the differences.

The study has three key findings, which we discuss below.

First, the overall HHSAF score and level did not change across hospitals. There are many barriers to the successful implementation of hand hygiene promotion and practice in LMICs, including a lack of resources to implement hand hygiene [19,20,21]. These barriers may have contributed to the lower mean hand hygiene scores reported in Sierra Leone and other LMICs and reflect the need to support these countries in strengthening their hand hygiene practices and promotion. 

In our setting, challenges such as a lack of dedicated budgets to support hand hygiene activities and a formalized patient engagement strategy were observed in May 2021 [12]. As alluded to earlier, we tried to address these gaps by introducing a hand hygiene improvement initiative in 2022 that included initial meetings with stakeholders at national and hospital levels. However, due to limited funding, follow-up meetings with stakeholders, including the hospital’s IPC leadership, were not held. Consequently, though patient engagement in hand hygiene remains a novel practice, the situation in many hospitals has not improved. Between May 2021 and April 2023, many hospitals have undergone a series of infrastructure and system modifications. Some hospitals have expanded bed capacity without corresponding increases in staffing. This increase in the number of hospital beds and the corresponding reduction in the staff capacity in April 2023 may have also contributed to these gaps. Understaffing has been documented to be associated with low hand hygiene compliance [22,23,24]. Moving forward, we plan to address these gaps by embedding a culture of quality and safety surrounding hand hygiene promotion in our hospitals. Again, we hope that the findings in this paper will act as a wake-up call for sustained implementation of hand hygiene improvement initiatives, including patient involvement in hand hygiene practices. Thus, we recommend the development of patient-friendly posters and other hand hygiene promotion resources in acute care settings to increase patient engagement in hand hygiene.

Second, in secondary hospitals, hand hygiene practice and promotion deteriorated from “intermediate or consolidation” to “basic” level. The decline may be partly due to infrastructure challenges (one hospital burned down and two were relocated due to ongoing renovations) or due to limited focus on post operational research interventions in secondary hospitals. Nonetheless, we observed that more secondary hospitals in April 2023 reported a continuous supply of alcohol-based hand rubs, and the number of hospitals with soap at each sink increased. Owing to the aftermath of the COVID-19 pandemic, more resources were put together by the government and partner organizations to increase local production of alcohol-based hand rubs, reflecting the increase in the excellent performance of the system change domain in these hospitals. The mean “system change” domain score in April 2023 was higher than that observed for tertiary hospitals but performed poorly in all other domains. Therefore, we will advocate focusing on efforts to address the gaps observed in the poorly performing domains.

Third, we observed an improvement in performance in the “system change” and “institutional safety climate” domains, but the “training and education” and “reminders in the workplace” domains worsened. Gaps such as lack of a dedicated budget to train healthcare workers, lack of a system for the recognition and utilization of hand hygiene role models or hand hygiene champions, and the inability of most hospitals to provide hand hygiene promotion resources may have affected the performance of most domains. Although these challenges are not unique to Sierra Leone, as they have been reported in Cambodia, Rwanda, and Nigeria, they call for a robust approach to advocate for budgetary allocation to support hand hygiene activities and other IPC practices in the country [21,25,26]. Furthermore, the low hand hygiene compliance rate reported in all the hospitals in May 2021 and April 2023 is a persistent and chronic problem affecting hand hygiene performance as low hand hygiene compliance rate has been previously reported in our hospitals [19,27].

Even though there is an improvement in the system change domain that could be attributed to the impact of COVID-19 on the development of hand hygiene infrastructure, we see a decline in other domains. The previous study was conducted in 2021; during this period, the incidence of COVID-19 cases was at its highest in Sierra Leone. Therefore, the hand hygiene scores were higher compared to our findings in 2023. For example, the training of healthcare workers and the availability of hand hygiene promotion posters declined in 2023. The impact of the COVID-19 pandemic on essential health service delivery is not unique to hand hygiene practices and promotion, as we have previously reported on the declines in HIV and TB services during the COVID-19 pandemic [28,29]. These facts underscore the importance of repeated assessment of hand hygiene practice and promotion at hospitals using an operational research approach to identify gaps in implementation, evaluate the sustainability of interventions, and use the evidence for action.

Despite the low mean HHSAF score reported in our study compared to that observed in the United States of America in 2011 (HHSAF score = 373), Greece in 2018 (HHSAF score = 289), and Italy in 2019 (HHSAF score = 332), the level remains at the same intermediate level (HHSAF Score 251–375) [30,31,32]. Notably, the mean hand hygiene scores in LMICs such as Cambodia (HHSAF score = 177), India (HHSAF score = 225), and Tanzania (HHSAF score = 187) were lower than those we report in May 2021 and April 2023 [21,33,34]. Sierra Leone experienced high-risk infectious disease epidemics such as the 2014–2016 Ebola outbreak, which led to the establishment of IPC governance structures in the country [10,35]. This could explain Sierra Leone’s good performance in hand hygiene promotion relative to other LMICs.

## 5. Conclusions

The implementation of recommendations of previous operational research in 2021 on hand hygiene practice promotion in the 13 public hospitals of the Western Area of Sierra Leone did not change the “intermediate” hand hygiene level. Furthermore, the deterioration in the hand hygiene level of secondary hospitals from an “intermediate” to “basic” level, the gaps in budgetary support, and the poor compliance of healthcare workers to hand hygiene practices will increase the spread of HAI and result in unnecessary mortality. In the short term, we will strengthen advocacy for our hospitals to dedicate a budget for hand hygiene activities, improve hand hygiene compliance and patient engagement, and train healthcare workers. Our medium-term plan is to adapt the HHSAF tool to our local context and train IPC practitioners on its use to inform monitoring and evaluation and future research.

## Figures and Tables

**Table 1:** Information and recommendations from the operational research conducted during Sierra Leone national SORT IT focused on antimicrobial resistance in 2021 [13].

Information/Findings (Key Messages)	Recommendations
Intermediate level of hand hygiene practices and promotion	Implement strategies to improve hand hygiene
No formalized patient engagement program	In addition to engaging healthcare workers on hand hygiene, we recommend the engagement of patients and relatives in hand hygiene promotion
No funding to support hand hygiene promotion, as many hospitals lack funding to support normal hospital activities	Hospital administrators should explore other sources of funding for sustaining local initiatives on hand hygiene practices and promotion
Limited hand hygiene resources for hand hygiene reminders in the workplace	We recommend hand hygiene posters at various places in hospitals, especially in hand hygiene stations
Lack of a system for the designation of hand hygiene champions or role models	Establish systems for recognizing hand hygiene champions and role models

HHSAF—hand hygiene self-assessment framework [9]. he recommendations are from an operational research study conducted during Sierra Leone national SORT IT focused on antimicrobial resistance in 2021 [13]. SORT IT—Structured Operational Research and Training Initiative.

**Table 2:** Dissemination details of the information and recommendations from the operational research conducted during Sierra Leone national SORT IT focused on antimicrobial resistance in 2021 [13,18].

To Whom	When	Where	Mode of Delivery *
Medical professionals and other healthcare workers	May to August 2022	Official WhatsApp group of the Ministry of Health and four professional groups	Video of lightening presentation
Two national engagement meetings	May and November 2022	Stakeholders from the Ministries of Health, Environment, and Agriculture	Technical PowerPoint presentation
Clinicians (around 80)	April 2022 and December 2022	Three tertiary hospitals in the Western Area of Sierra Leone	Technical PowerPoint presentation
Hospital management of one tertiary hospital	October 2022	One of the tertiary hospitals	Handout [18] and Elevator pitch

SORT IT—Structured Operational Research and Training Initiative. * these advocacy tools were developed during module 4 (Communicating research findings) of Sierra Leone national SORT IT, which is focused on antimicrobial resistance.

**Table 3:** List of the decisions taken during the dissemination of findings/recommendations from the operational research conducted during Sierra Leone national SORT IT * focused on antimicrobial resistance in 2021 [13].

Decisions	Action Status **	Details of Action (When and What)
Strengthen the distribution of hand hygiene posters and alcohol-based hand rub	Ongoing	By working with the national IPC program, a regular monthly supply of alcohol-based hand rub was maintained
Strengthen training of healthcare workers on hand hygiene	Ongoing	Orientation of new staff employed by the hospital on hand hygiene at any intake
Advocacy for budgetary support for hand hygiene	Ongoing	During the national engagement meeting in November 2023
Advocacy to include hand hygiene in hospital improvement programs supported by partners	Completed	Hand hygiene was included in the implementation of quality IPC services supported by an implementing partner in three hospitals in December 2022
Develop a policy for patient engagement on IPC	Ongoing	A written policy that shows how patients and relatives should be involved in hand hygiene practice and promotion is currently under development

* SORT IT—Structured Operational Research and Training Initiative and IPC—Infection prevention and control. ** as of January 2023.

**Table 4:** Characteristics of the 13 public hospitals in Western Area of Sierra Leone, before (May 2021) and after (April 2023) the implementation of the recommendations from operational research *.

Hospital Characteristics	Before		After	
	*n*	(%)	*n*	(%)
**Total**	13	(100)	13	(100)
**Bed Capacity**				
≤50	3	(23.1)	3	(23.1)
51–100	4	(30.8)	4	(30.8)
101–150	1	(7.7)	2	(15.4)
151–200	2	(15.4)	2	(15.4)
>200	3	(23.1)	2	(15.4)
**Mean Bed Capacity**		111		132
**Staff Capacity**				
≤200	5	(38.5)	6	(46.2)
201–400	4	(30.8)	2	(15.4)
400–600	3	(23.1)	4	(30.8)
≥601	1	(7.7)	1	(7.7)
**Mean Staff Capacity**		277		241
**Units/Wards**				
<10	1	(7.7)	5	(38.5)
10–20	8	(61.5)	6	(46.2)
>20	4	(30.8)	2	(15.4)
Level of care				
Tertiary	7	(53.8)	7	(53.8)
Secondary	6	(46.2)	6	(46.2)

* The recommendations are from an operational research study conducted during Sierra Leone national SORT IT focused on antimicrobial resistance in 2021 [13].

**Table 5:** Mean scores of hand hygiene practice and promotion assessment (using HHSAF) in the 13 public hospitals in Western Area of Sierra Leone, before (May 2021) and after (March–April 2023) the implementation of the recommendations from operational research * [13].

Score Using HHSAF	Before		After	
	Mean	(SD)	Mean	(SD)
System change	52.3	(17.3)	72.7	(10.7)
Training and education	66.9	(17.4)	50.0	(17.6)
Evaluation and feedback	54.0	(13.4)	53.9	(11.6)
Reminders in the workplace	50.0	(18.1)	43.2	(16.0)
Institutional safety climate	46.9	(19.6)	58.5	(14.6)

HHSAF—hand hygiene self-assessment framework [9]; and SD—standard deviation. * The recommendations are from operational research conducted during Sierra Leone national SORT IT focused on antimicrobial resistance in 2021 [13].

**Table 6:** Distribution of total and domain-wise hand hygiene practice and promotion assessment (using HHSAF) across the 13 public hospitals in Western Area of Sierra Leone, before (May 2021) and after (March–April 2023) the implementation of the recommendations from operational research * [13].

		Before						After					
Hospital Type	Hospital	SC	TE	EF	RW	ISC	HH Level	SC	TE	EF	RW	ISC	HH Level
**Secondary**	S1	*35*	80	48	55	50	I/268	75	70	60	20	70	I/295
	S2	40	*35*	58	50	*30*	B/213	75	35	35	15	50	B/210
	S3	55	80	65	50	65	I/315	80	35	40	25	50	B/230
	S4	80	50	45	70	*30*	I/275	75	30	45	45	55	B/250
	S5	50	70	60	45	65	I/290	85	50	65	48	65	I/313
	S6	75	50	68	63	50	I/305	70	35	45	25	25	B/200
**Tertiary**	T1	40	100	75	*33*	*35*	I/283	70	80	60	50	85	I/345
	T2	65	75	65	*25*	65	I/295	80	35	65	50	65	I/295
	T3	55	65	*35*	*15*	40	B/210	75	40	70	60	60	I/305
	T4	50	75	53	*38*	40	I/255	70	60	55	50	55	I/290
	T5	*30*	55	*35*	68	*25*	B/213	75	70	65	60	55	I/325
	T6	*30*	55	*35*	68	*25*	B/213	40	40		58	50	B/228
	T7	75	80	60	70	90	I/375	75	70	55	55	75	I/330

HHSAF = hand hygiene self-assessment framework [9], SC = system change; TE = training and education; EF = evaluation and feedback; RW = reminders in the workplace; ISC = institutional safety climate; HH = hand hygiene practice and promotion; I = intermediate; B = basic; S1 to S6 = secondary hospitals 1 to 6 and T1 to T7 = tertiary hospitals 1 to 7; green = excellent performance (>70); yellow = good (51–70); orange = poor (35–50); red = very poor (<35); * the recommendations are from an operational research conducted during Sierra Leone national SORT IT focused on antimicrobial resistance in 2021 [13].

## Data Availability

The data used in this study are available at the University of Sierra Leone repository and will be available on request to the corresponding author (M.N.K.).

## Supplementary Materials

The following supporting information can be downloaded at https://www.mdpi.com/article/10.3390/tropicalmed8110486/s1. Table S1: Interpretation of the total score and domain-wise score from the WHO HHSAF tool [9]. Table S2: Total number of hospitals (N) with a positive response for specific indicators under the five domains of HHSAF tool in Western Area of Sierra Leone before (May 2021) and after (March–April 2023) the implementation of the recommendations from operational research.

## Open Access Statement

In accordance with WHO’s open-access publication policy for all work funded by WHO or authored/co-authored by WHO staff members, WHO retains the copyright of this publication through a Creative Commons Attribution IGO license (http://creativecommons.org/licenses/by/3.0/igo/legalcode, accessed on the 14 July 2023) which permits unrestricted use, distribution, and reproduction in any medium provided the original work is properly cited.

## References

[1] World Health Organization (2011). Report on the Burden of Endemic Health Care-Associated Infection Worldwide Clean Care Is Safer Care.

[2] Nejad S.B., Allegranzi B., Syed S.B., Ellisc B., Pittetd D. (2011). Health-Care-Associated Infection in Africa: A Systematic Review. Bull. World Health Organ..

[3] Jia H., Li L., Li W., Hou T., Ma H., Yang Y., Wu A., Liu Y., Wen J., Yang H. (2019). Impact of Healthcare-Associated Infections on Length of Stay: A Study in 68 Hospitals in China. BioMed Res. Int..

[4] Serra-Burriel M., Keys M., Campillo-Artero C., Agodi A., Barchitta M., Gikas A., Palos C., López-Casasnovas G. (2020). Impact of Multi-Drug Resistant Bacteria on Economic and Clinical Outcomes of Healthcare-Associated Infections in Adults: Systematic Review and Meta-Analysis. PLoS ONE.

[5] Alp E., Damani N. (2015). Healthcare-Associated Infections in Intensive Care Units: Epidemiology and Infection Control in Low-to-Middle Income Countries. J. Infect. Dev. Ctries..

[6] Rothe C., Schlaich C., Thompson S. (2013). Healthcare-Associated Infections in Sub-Saharan Africa. J. Hosp. Infect..

[7] Kingston L., O’Connell N.H., Dunne C.P. (2016). Hand Hygiene-Related Clinical Trials Reported since 2010: A Systematic Review. J. Hosp. Infect..

[8] Loftus M.J., Guitart C., Tartari E., Stewardson A.J., Amer F., Bellissimo-Rodrigues F., Lee Y.F., Mehtar S., Sithole B.L., Pittet D. (2019). Hand Hygiene in Low- and Middle-Income Countries. Int. J. Infect. Dis..

[9] World Health Organization (2010). Hand Hygiene Self-Assessment Framework 2010 Introduction and User Instructions.

[10] Ministry of Health and Sanitation, Government of the Republic of Sierral Leone (2016). National Infection Prevention and Control Guidelines.

[11] Ministry of Health and Sanitation, Government of Sierra Leone (2015). Ebola Viral Disease Situation Report.

[12] Special Programme for Research & Training in Tropical Diseases (TDR) (2023). AMR-SORT IT 2022 Annual Report. Progress, Achievements, Challenges.

[13] Lakoh S., Maruta A., Kallon C., Deen G.F., Russell J.B.W., Fofanah B.D., Kamara I.F., Kanu J.S., Kamara D., Molleh B. (2022). How Well Are Hand Hygiene Practices and Promotion Implemented in Sierra Leone? A Cross-Sectional Study in 13 Public Hospitals. Int. J. Environ. Res. Public Health.

[14] Statistics Sierra Leone (2016). 2015 Population and Housing Census. Summary of Final Results.

[15] International Monetary Fund Sierra Leone. https://www.imf.org/en/Countries/SLE#ataglance.

[16] The World Bank Sierra Leone. https://data.worldbank.org/country/SL.

[17] Statistics Sierra Leone, United Nations Children’s Fund (UNICEF) (2017). Sierra Leone Multuple Indicator Cluster Survey 2017.

[18] Special Programme for Research & Training in Tropical Diseases (TDR) AMR-SORT IT Evidence Summaries: Communicating Research Findings. https://tdr.who.int/activities/sort-it-operational-research-and-training/communicating-research-findings.

[19] Lakoh S., Firima E., Williams C.E.E., Conteh S.K., Jalloh M.B., Sheku M.G., Adekanmbi O., Sevalie S., Kamara S.A., Kamara M.A.S. (2021). An Intra-COVID-19 Assessment of Hand Hygiene Facility, Policy and Staff Compliance in Two Hospitals in Sierra Leone: Is There a Difference between Regional and Capital City Hospitals?. Trop. Med. Infect. Dis..

[20] Ataiyero Y., Dyson J., Graham M. (2019). Barriers to Hand Hygiene Practices among Health Care Workers in Sub-Saharan African Countries: A Narrative Review. Am. J. Infect. Control.

[21] An B., Yang S.J. (2020). The Evaluation of a Multimodal Hand Hygiene Improvement Strategy in Cambodian Hospitals. J. Infect. Dev. Ctries..

[22] Pittet D. (2001). Improving Adherence to Hand Hygiene Practice: A Multidisciplinary Approach. Emerg. Infect. Dis..

[23] Mathur P. (2011). Hand Hygiene: Back to the Basics of Infection Control. Indian J. Med. Res..

[24] Muller M.P., Carter E., Siddiqui N., Larson E. (2015). Hand Hygiene Compliance in an Emergency Department: The Effect of Crowding. Acad. Emerg. Med..

[25] Uneke C.J., Ndukwe C.D., Oyibo P.G., Nwakpu K.O., Nnabu R.C., Prasopa-Plaizier N. (2014). Promotion of Hand Hygiene Strengthening Initiative in a Nigerian Teaching Hospital: Implication for Improved Patient Safety in Low-Income Health Facilities. Braz. J. Infect. Dis..

[26] Holmen I.C., Seneza C., Nyiranzayisaba B., Nyiringabo V., Bienfait M., Safdar N. (2016). Improving Hand Hygiene Practices in a Rural Hospital in Sub-Saharan Africa. Infect. Control Hosp. Epidemiol..

[27] Kamara G.N., Sevalie S., Molleh B., Koroma Z., Kallon C., Maruta A., Kamara I.F., Kanu J.S., Campbell J.S.O., Shewade H.D. (2022). Hand Hygiene Compliance at Two Tertiary Hospitals in Freetown, Sierra Leone, in 2021: A Cross-Sectional Study. Int. J. Environ. Res. Public Health.

[28] Lakoh S., Bangura M.M., Adekanmbi O., Barrie U., Jiba D.F., Kamara M.N., Sesay D., Jalloh A.T., Deen G.F., Russell J.B.W. (2023). Impact of COVID-19 on the Utilization of HIV Testing and Linkage Services in Sierra Leone: Experience from Three Public Health Facilities in Freetown. AIDS Behav..

[29] Lakoh S., Jiba D.F., Baldeh M., Adekanmbi O., Barrie U., Seisay A.L., Deen G.F., Salata R.A., Yendewa G.A. (2021). Impact of COVID-19 on Tuberculosis Case Detection and Treatment Outcomes in Sierra Leone. Trop. Med. Infect. Dis..

[30] Kritsotakis E.I., Astrinaki E., Messaritaki A., Gikas A. (2018). Implementation of Multimodal Infection Control and Hand Hygiene Strategies in Acute-Care Hospitals in Greece: A Cross-Sectional Benchmarking Survey. Am. J. Infect. Control.

[31] Allegranzi B., Conway L., Larson E., Pittet D. (2014). Status of the Implementation of the World Health Organization Multimodal Hand Hygiene Strategy in United States of America Health Care Facilities. Am. J. Infect. Control..

[32] Bert F., Giacomelli S., Ceresetti D., Zotti C.M. (2019). World Health Organization Framework: Multimodal Hand Hygiene Strategy in Piedmont (Italy) Health Care Facilities. J. Patient Saf..

[33] Wiedenmayer K., Msamba V.-S., Chilunda F., Kiologwe J.C., Seni J. (2020). Impact of Hand Hygiene Intervention: A Comparative Study in Health Care Facilities in Dodoma Region, Tanzania Using WHO Methodology. Antimicrob. Resist. Infect. Control.

[34] Sharma R., Sharma M., Koushal V. (2014). Compliance to Hand Hygiene World Health Organization Guidelines in Hospital Care. Int. J. Prev. Med..

[35] Omilabu S.A., Salu O.B., Oke B.O., James A.B. (2016). The West African Ebola Virus Disease Epidemic 2014–2015: A Commissioned Review. Niger. Postgrad. Med. J..

