# Supplementary material for: Hand Hygiene Practices and Promotion in Public Hospitals in Western Sierra Leone: Changes Following Operational Research in 2021
Source: Trop Med Infect Dis. 2023 Oct 27;8(11):486. doi: 10.3390/tropicalmed8110486 (PMC10674222; doi:10.3390/tropicalmed8110486)
Supplement: Supplementary file 1 [file tropicalmed-08-00486-s001.zip › tropicalmed-2475670-supplementary.pdf]

S1 Annex. Interpretation of the total score and domain wise score from the WHO HHSAF tool (WHO, 2010)

Overall Assessment of hand hygiene practice and promotion based on total score

Color code	Total Score	Level	Definition
(red)	0-125	Inadequate	Indicates insufficient hand hygiene practices and promotion, and requires significant improvement
(orange)	126-250	Basic	Indicates that some measures are in place but not satisfactory and therefore requires further improvement
(yellow)	251-375	Intermediate or consolidate	Indicates appropriate hand hygiene promotion strategies and improvements in hand hygiene practices, but requires long-term planning to ensure continual improvement and progress
(green)	376-500	Advance or embedding	Indicates sustained hand hygiene promotion and practice as well as a quality and safety culture surrounding hand hygiene promotion within the organization

Level of the domains

Color code	Domain score*	Level
(green)	>70	Excellent
(yellow)	51-70	Good
(orange)	35-50	Poor
(red)	<35	Very poor

WHO – world health organization, HHSAF – hand hygiene self-assessment framework, *Cut offs used for domain wise score are author's arbitrary cut offs

S2 Annex. Total number of hospitals(N) with a positive response for specific indicators under the five domains of HHSAF tool in Western Area of Sierra Leone, before (May 2021) and after (March-April 2023) the implementation of the recommendations from operational research* (WHO, 2010)-appendix 2.

**Question / Indicator**	**Before N (%)**	**After N(%)**
**1. System Change**		
1.1. Are alcohol based handrub available facility-wide with continuous supply at each point of care (with efficacy and tolerability proven)?	6(46)	12(92)
1.2. Is the sink to bed ratio at least 1:10 facility-wide and 1:1 in isolation rooms and in intensive care units	4(31)	3(23)
1.3. Is there a continuous supply of clean, running water?	8(61)	9(69)
1.4. Is soap available at each sink?	10(77)	13(100)
1.5. Are single-use towels available at each sink?	0(0)	0(0)
1.6. IS there a dedicated budget for continuous procurement of hand hygiene products	0(0)	3(23)
1.7. Is there a realistic plan in place to improve the infrastructure in your healthcare facility?	0(0)	4(31)
**2. Training and Education**		
2.1. Are there mandatory training for all professional categories at commencement of employment, then ongoing regular training (at least annually)	6(46)	4(31)
2.2. Is a process in place to confirm that all healthcare workers complete this training?	10(77)	7(54)
2.3. Is a professional with adequate skills to serve as trainer for hand hygiene educational programs active within the healthcare facility?	12(92)	13(100)
2.4. Is a system in place for training and validation of hand hygiene compliance observers?	12(92)	10(77)

2.5. Is there a dedicated budget that allows for hand hygiene training?	1(8)	2(15)
**3. Evaluation and Feedback**		
3.1. Are regular (at lease annual) ward-based audits undertaken to assess the availability of handrub, soap, single-use towels and other hand hygiene resources?	2(15)	0(0)
3.2. Is healthcare worker knowledge of the indications for hand hygiene assessed at least annually?	11(85)	8(62)
3.3. Is healthcare worker knowledge of the correct technique for hand hygiene assessed at least annually?	10(77)	8(62)
3.4. Is consumption of alcohol-based handrub monitored regularly (at least every 3 months)?	9(69)	0(0)
3.5. Is consumption of soap monitored regularly (at least every 3 months)?	9(69)	0(0)
3.6. Is alcohol-based handrub consumption at least 20 L per 1000 patient-days?	3(23)	0(00
3.7. Is direct observation of hand hygiene compliance performed every 3 months or more often using the WHO hand hygiene observation tools (or similar technique)?	11(85)	12(92)
3.8. Was the maximum (71-80%) hand hygiene compliance rate according to the WHO hand hygiene observation tool (or similar technique) in your facility?	0(0)	0(0)
**4. Reminders in the workplace**		
4.1. Is poster explaining the indications for hand hygiene displayed in all wards/treatment areas	9(69)	4(31)
4.2. Is poster explaining the correct use of hand rub displayed in all wards/treatment areas	8(62)	2(15)
4.3. Is poster explaining correct handwashing technique displayed at every sink in all wards/treatment areas	8(62)	6(46)
4.4. Is there a systematic audit of all posters for evidence of damage, with replacement as required?	0(0)	4(31)
4.5. Is hand hygiene promotion undertaken by displaying and regularly updating posters other than those mentioned above?	3(23)	5(38)
4.6. Are hand hygiene information leaflets available in all the wards?	0(0)	0(0)

4.7. Are other workplace reminders located throughout the facility? (e.g., hand hygiene campaign screensavers badges, stickers, etc)	2(15)	1(8)
**5. Institutional Safety Climate for Hand Hygiene**		
5.1. Is hand hygiene team that is dedicated to the promotion and implementation of optimal hand hygiene practice established in your facility?	12(92)	12(92)
5.2. Does this hand hygiene team meet on a regular basis (at least annually)?	10(75)	7(54)
5.3. Does this hand hygiene team have dedicated time to conduct hand hygiene promotion?	7(54)	10(77)
Are systems for designation of hand hygiene champions from all disciplines in place?	7(54)	3(23)
5.4. Are systems for recognition and utilization of hand hygiene role models from all disciplines in place?	5(39)	1(8)
5.5. Are patients informed about the importance of hand hygiene?	7(54)	10(77)
5.6. Has a formalized program of patient engagement been undertaken?	3(23)	5(39)

HHSAF - hand hygiene self-assessment framework [9], * the recommendations are from an operational research conducted during Sierra Leone national SORT IT focused on antimicrobial resistance in 2021 [13]
